# Bolus Injection of Liraglutide Raises Plasma Glucose in Normal Rats by Activating Glucagon-like Peptide 1 Receptor in the Brain

**DOI:** 10.3390/ph15070904

**Published:** 2022-07-21

**Authors:** Chia-Chen Hsu, Juei-Tang Cheng, Ping Hao Hsu, Yingxiao Li, Kai-Chun Cheng

**Affiliations:** 1 Graduate Institute of Gerontology and Health Care Management, Chang Gung University of Science and Technology, Taoyuan City 33303, Taiwan; eardoctorhsu@yahoo.com.tw; 2 Department of Otorhinolaryngology, Taipei City Hospital, Taipei City 10341, Taiwan; 3 Department of Exercise and Health Sciences, University of Taipei, Taipei City 11153, Taiwan; 4 Department of Medical Research, Chi-Mei Medical Center, Tainan City 71004, Taiwan; jtceng5503@gmail.com; 5 School of Medicine, Chung Shan Medical University, Taichung City 40201, Taiwan; andy30817@gmail.com; 6 Department of Nursing, Tzu Chi University of Science and Technology, Hualien 970302, Taiwan; 7 Department of Pharmacy, College of Pharmacy, Tajen University, Pingtung 90741, Taiwan

**Keywords:** liraglutide, Exendin-4, GLP-1 receptor, hypothalamic-pituitary-adrenal (HPA) axis, feeding behavior, rats

## Abstract

Diabetes is commonly treated with glucagon-like peptide-1 receptor (GLP-1R) agonists including liraglutide and others. However, liraglutide was found to raise plasma glucose levels in normal rats. The current study aims to determine how liraglutide causes this contentious condition in rats, both normal and diabetic. An adrenalectomy was performed to investigate the relationship between steroid hormone and liraglutide. To investigate the effect of central liraglutide infusion on blood glucose in rats, rats were intracerebroventricularly administrated with liraglutide with or without HPA axis inhibitors such as berberine and dexamethasone. The results showed that a single injection of liraglutide caused a temporary increase in blood glucose in healthy rats. Another GLP-1R agonist, Exendin-4 (Ex-4), increased blood sugar in a manner similar to that of liraglutide. The effects of liraglutide were also blocked by guanethidine pretreatment and vanished in normal rats with adrenalectomy. Additionally, central infusion of liraglutide via intracerebroventricular (icv) injection into normal rats also causes a temporary increase in blood glucose that was blocked by GLP-1R antagonists or the inhibitors such as berberine and dexamethasone. Similarly, central liraglutide treatment causes temporary increases in plasma glucose, adrenocorticotropic hormone (ACTH), and cortisol levels, which were reversed by inhibitors for the hypothalamic-pituitary-adrenal (HPA) axis. In normal rats, the temporary glucose-increasing effect of liraglutide was gradually eliminated during consecutive daily treatments, indicating tolerance formation. Additionally, liraglutide and Ex-4 cross-tolerance was also discovered in normal rats. Liraglutide was more effective in diabetic rats than in normal rats in activating GLP-1R gene expression in the isolated adrenal gland. Interestingly, the effect of liraglutide on glycemic control varied depending on whether the rats were diabetic or not. In normal rats, bolus injection of liraglutide, such as Ex-4, may stimulate the HPA axis, resulting in hyperglycemia. The cross-tolerance of liraglutide and Ex-4 provided a novel perspective on GLP-1R activation.

## 1. Introduction

Liraglutide is a known fatty acid acylated analogue of GLP-1 with an efficacy for 24 h/day. It shares 97% homology with endogenous GLP-1 and is better than exenatide which has 57% homology. After binding with GLP-1 receptor (GLP-1R), a G-protein-coupled receptor (GPCR) located on the cell membrane, liraglutide is introduced to activate through an increase in cyclic adenosine monophosphate (cAMP). Liraglutide can increase glucose-dependent insulin secretion from pancreatic tissues, lowering plasma glucose levels with little risk of hypoglycemia in both humans and animals [1,2]. It may also inhibit glucagon secretion through GLP-1R activation to lower glucose production in the liver. Additionally, liraglutide has been identified to enhance pancreatic beta cell mass and function after promoting replication and reducing apoptosis [3]. Therefore, liraglutide is widely used in the treatment of type 2 diabetes (T2DM). On the other hand, liraglutide is also known to delay gastric emptying and acid secretion, resulting in lower post-prandial hyperglycemia and increased satiety [4]. In clinics, GLP-1R agonist such as exenatide (twice daily) has been applied to treat T2DM since 2007. Compounds with a longer action (once daily, q.d.; or once weekly, q.w.) and/or an increased glycemic regulated efficacy in addition to the lowering of obesity have been developed. Liraglutide is dosed once daily in clinical practice. However, the injectable therapy is recommended only in diabetic cases with a failure of oral medications [5]. Additionally, these peptide agonists (all for subcutaneous injection, except the oral semaglutide) have been compared in cardiovascular outcomes. It has been indicated that liraglutide, semaglutide, albiglutide, and dulaglutide may reduce the time to the first cardiovascular adverse events, while liraglutide can reduce cardiovascular and all-cause mortality [6]. Therefore, liraglutide is considered as a useful agent in therapeutics of T2DM.

Recent findings have demonstrated that GLP-1 has an effect on the hypothalamic paraventricular nucleus, which connects GLP-1 to corticotropin-releasing hormone neurons [7]. In rodents, acute central and peripheral administration of GLP-1, Exendin-4 (Ex-4) or liraglutide has been shown to activate the hypothalamic–pituitary–adrenal (HPA) axis by increasing ACTH and cortisol levels in the blood [8,9]. In animal studies, a single dose of the GLP-1 ligand Ex-4 was shown to cause acute hyperglycemia in normal rats [10]. It appears to contradict the widely held belief about the effects of GLP-1R agonists. Liraglutide, in fact, has been shown to reduce hyperglycemia in type 1 diabetic rats [11]. Therefore, it is especially intriguing to see if this is also produced by another agonist in addition to Ex-4.

The main aim of the current study was to investigate the central and peripheral effects of liraglutide in rats under normal physiological conditions and diabetic conditions. In addition, the effects of GLP-1R agonists on the HPA axis activity were compared via different drugs and treatments. We also investigated the possible mechanism(s) underlying the variations.

## 2. Results

### 2.1. Acute Effects of Liraglutide on Plasma Glucose in Normal Rats

**Figure 1:**
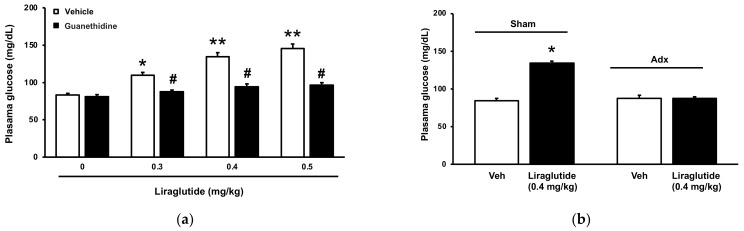
Liraglutide-induced acute hyperglycemia in healthy rats; (**a**) The dose–response curve (open column) of liraglutide that is reduced by guanethidine (closed column). IP injection of guanethidine (30 mg/kg) for 60 min prior to liraglutide treatment. (**b**) Liraglutide-induced hyperglycemia in rats receiving adrenalectomy or sham-operation (Sham). Hyperglycemia by liraglutide disappeared in adrenalectomized rats but was observed in sham-operated rats. The data are shown as means ± SEM (*n* = 8). * *p* < 0.05, ** *p* < 0.01 vs. normal control group; # *p* < 0.05 vs. vehicle-treated group.

Bolus injection of liraglutide significantly increased acute plasma glucose levels in normal rats (Figure 1a). Guanethidine is a sympatholytic medication that reduces norepinephrine levels in postganglionic sympathetic nerve endings [12]. The current findings show that guanethidine inhibits the action of liraglutide in normal rats (Figure 1a). Furthermore, we performed the bilateral adrenalectomy in rats, as described previously [11]. Rats receiving a sham-operation were used as the control. Compared to the sham-operated group, the adrenalectomy group did not demonstrate an increase in glucose induced by liraglutide (Figure 1b).

### 2.2. Acute Hyperglycemia in Rats Received Injection of Liraglutide into Brain

**Figure 2:**
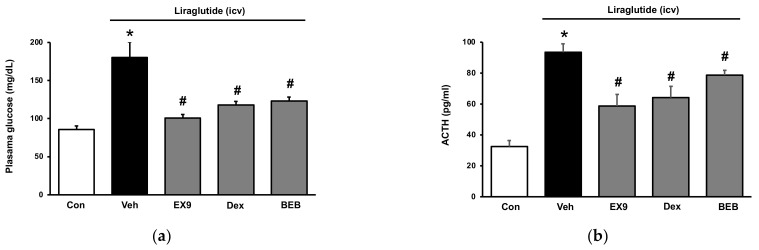

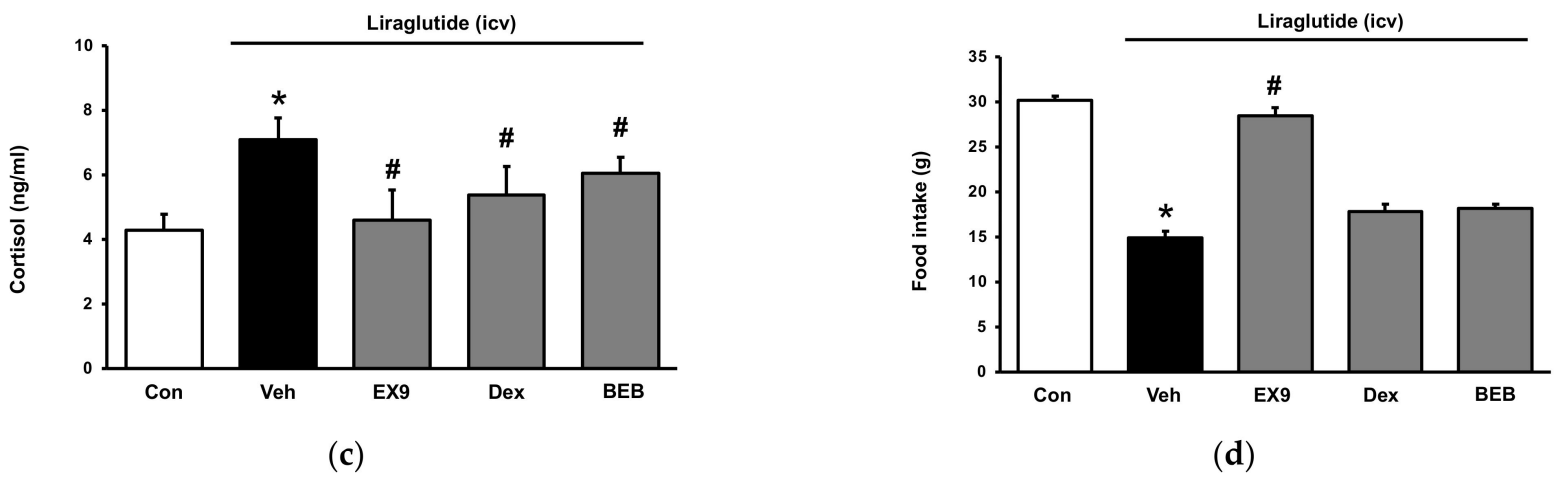
Effect of liraglutide on hypothalamic-pituitary-adrenal (HPA) axis in healthy rats. Liraglutide was injected into the brain directly and changes induced by liraglutide were compared with pretreatment of inhibitors including EX-9 (i.c.v.), berberine (BER, 200 mg/kg, i.p.) and dexamethasone (DEX, 5 μg/kg, s.c.). Each inhibitor was pretreated by peripheral administration for 60 min, except EX-9, which was pretreated for 30 min by injection into the brain. (**a**) Changes in plasma glucose in rats; (**b**) Changes in plasma ACTH in rats; (**c**) Changes in plasma cortisol in rats; (**d**) Changes in feeding behavior in rats. The data are shown as means ± SEM (*n* = 8). * *p* < 0.05 vs. normal control group; # *p* < 0.05 vs. vehicle (Veh)-treated group.

In healthy rats, the effects of a single ICV injection of liraglutide on blood glucose regulation were studied. Liraglutide significantly increased plasma glucose levels shortly after administration. (Figure 2a). This effect was mitigated by ICV pretreatment of Ex9 at the dose required to block GLP-1R [6].

Berberine has been shown in rats to inhibit the HPA axis while increasing skeletal muscle GLUT4 expression [13]. Additionally, dexamethasone has similar effects in that it can quickly block stress induced HPA activity [14]. In the present study, oral intake of berberine (200 mg/kg) [13] inhibited hyperglycemia induced by liraglutide. Subcutaneous injection of dexamethasone at the effective dose (5 μg/kg) [15] also attenuated a hyperglycemic response to liraglutide. In addition, liraglutide induced an elevation of ACTH (Figure 2b) and cortisol (Figure 2c) in normal rats after ICV injection. Ex9 reduced the protective effects of liraglutide. Berberine and dexamethasone, both of which inhibit the HPA-axis pathway, reduced liraglutide-induced increases in plasma ACTH and cortisol levels in rats. However, ICV injection of Ex9 reversed liraglutide induced anorexia but not berberine or dexamethasone (Figure 2d).

### 2.3. Tolerance of Hyperglycemia-Induced by Liraglutide in Normal Rats

**Figure 3:**
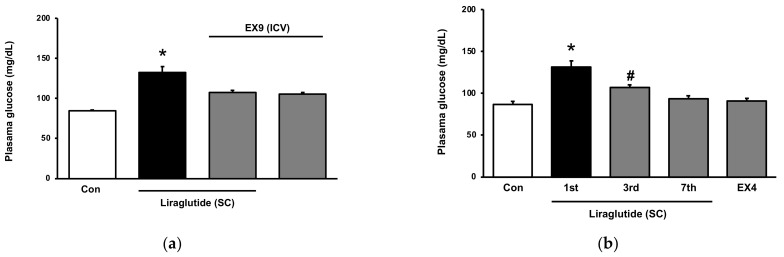
Cross-tolerance of hyperglycemia between liraglutide and Exendin-4 (Ex-4) in normal rats. (**a**) Hyperglycemia by peripheral injection of liraglutide (SC) was blocked by EX-9 in the brain (ICV); (**b**) Tolerance of hyperglycemia by liraglutide observed in normal rats received a repeated daily injection for one week. Another agonist of GLP-1R Ex-4 also failed to induce hyperglycemia in normal rats with tolerance to liraglutide. The data are shown as means ± SEM (*n* = 8). * *p* < 0.05 vs. normal control group; # *p* < 0.05 vs. first day.

In normal rats, hyperglycemia induced by liraglutide decreased gradually during repeated daily treatment. After 7 days and more, this response disappeared and liraglutide induced a mild reduction in plasma glucose 10 days later. This suggests tolerance of liraglutide. Similarly, hyperglycemia induced by Ex-4 also showed tolerance in the same manner after repeated treatment for 5 days and more in healthy rats. Notably, Ex-4 failed to increase plasma glucose in normal rats with tolerance to liraglutide (Figure 3b). A cross-tolerance between Ex-4 and liraglutide was observed in normal rats. Therefore, activation of HPA axis by liraglutide in normal rats (Figure 3a) seems to be easily tolerated after a repeated treatment. The results indicate a cross-tolerance between Ext-4 and liraglutide in normal rats. Therefore, the temporary hyperglycemia induced by liraglutide in normal rats appears to be well mitigated after repeated administration.

### 2.4. Role of Receptor Sensitivity in the Dual Effects of Liraglutide In Vivo

**Figure 4:**
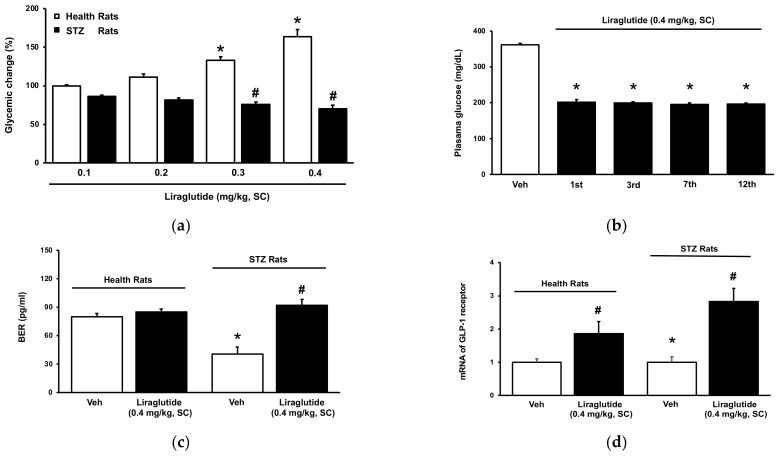
Influence of liraglutide in type-1 diabetic rats. (**a**) Dose-dependent effects of liraglutide on plasma glucose in diabetic rats were different with that in normal rats. Glycemic change was calculated by the percentage change in resulted plasma glucose prior; (**b**) Tolerance to liraglutide-induced hypoglycemia was not observed in diabetic rats and hypoglycemic response to liraglutide produced in diabetic rats receiving daily injection; (**c**) Effects of liraglutide on adrenal gland isolated from diabetic rats were different with that from normal rats. Beta-endorphin released by liraglutide as the functions of GLP-1R activation was observed in adrenal glands isolated from diabetic rats only; (**d**) The mRNA level of GLP-1R in adrenal glands between normal and diabetic rats. GLP-1R expression promoted by liraglutide in adrenal glands was higher in diabetic rats than normal rats. Thus, liraglutide promotes the mRNA level of GLP-1R in adrenal glands isolated from diabetic rats only. The data are shown as means ± SEM (*n* = 8). * *p* < 0.05 vs. low dose of liraglutide (**a**), vs. vehicle (Veh)-treated group (**b**), vs. normal rats (**c**) vs. normal group (**d**); # *p* < 0.05 vs. low dose of liraglutide (**a**), vs. vehicle (Veh)-treated group (**c**,**d**).

Different doses of liraglutide were given to normal and diabetic rats to compare their effects. Under the same conditions, the diabetic group’s blood glucose levels were significantly lower than those of the control group (Figure 4a). Furthermore, the diabetic rats were discovered to be sensitive to the glucose-lowering effect of liraglutide, which was demonstrated at a low dose (0.1 mg/kg). In the diabetic group, the glucose-lowering effect of liraglutide was consistently present during the daily repeated treatment (Figure 4b). The secretion of beta-endorphin by liraglutide was observed in the isolated adrenal glands of diabetic rats but not in those of healthy rats (Figure 4c). Similarly, the mRNA level of GLP-1R was promoted by liraglutide in adrenal glands isolated from diabetic rats but not in those from normal rats (Figure 4d). Liraglutide appears to be more effective in regulating glucose, increasing beta-endorphin secretion and increasing GLP-1 receptor expression in diabetics than that in non-diabetics.

## 3. Discussion

A bolus injection of liraglutide was found to raise plasma glucose levels in normal rats, primarily by activating the hypothalamic-pituitary-adrenal (HPA) axis in the brain. It is easily tolerated after daily repeated treatment, similar to the effect of Ex-4, another GLP-1 analogue. Notably, we discovered a previously unknown cross-tolerance between liraglutide and Ex-4.

Ex-4 has been shown in rats to cause hyperglycemia, most likely as a result of sympathetic tone activation [10]. Later, it was reported to cause HPA axis activation in rats [8,16], as well as GLP-1 innervation in the hypothalamus [15]. However, it was considered a nonspecific effect of Ex-4. In the current study, liraglutide had the same effect on normal rats as Ex-4. As a result of the cross-tolerance between Ex-4 and liraglutide, it appears that the general effect of the GLP-1R agonist, rather than a pleiotropic effect of Ex-4, is possible. More data is needed to back this up, including cross-tolerance between more GLP-1R agonists used in a clinical setting.

A similar trend was observed in the Ex-4-treated group, which was consistent with previous research [10,17]. These findings imply that GLP-1R activation may cause sympathetic nervous system activation, resulting in an acute increase in blood glucose levels. GLP-1R is regulated by vagal afferent neurons that innervate the gastrointestinal tract, including the hepatoportal region, and by nodose ganglion neurons [18]. Furthermore, GLP-1R agonists have been linked to the activation of the HPA axis in rats [8,16] and the innervation of GLP-1 in the hypothalamus [11].

Furthermore, liraglutide-induced hyperglycemia is as easily tolerated as Ex-4 in normal rats. The hyperglycemic response to liraglutide was the same as Ex-4 gradually decreasing after repeated treatment or prolonged administration, indicating that sympathetic nervous system activation by GLP-1R agonist diminishes over time. In normal rats, both were restored to normal after one week of repeated treatment. Liraglutide stimulates secretion of ACTH and cortisol in normal rats, consistent with the effects of Ex-4, especially after direct injection into the brain. Therefore, liraglutide is the 2nd ligand of GLP-1R to increase plasma glucose in normal rats such as Ex-4.

Notably, rats showed cross-tolerance between liraglutide and Ex-4. This does not appear to be due to receptor downregulation because the anorexia induced by liraglutide was not altered after the same repeated treatment. Previous studies [19,20] found that GLP-1 analogues significantly reduce body weight and food intake (anorexia). Although it may not appear to be simple, there is no doubt that the GLP-1R regulates both food intake and feeding behavior (hunger-driven feeding, food hedonic value, and food motivation) [18]. Liraglutide has been shown to activate GLP-1R in the hippocampus [21], resulting in a decrease in food intake. Thus, it appears that tolerance of the HPA axis occurred in the hypothalamus but not in the hippocampus [16]. As a result, the previously unknown cross-tolerance was produced between liraglutide and Ex-4 in normal rats. However, the mechanisms(s) for this result must be investigated in advance.

The adrenal gland plays an important role in the regulation of the HPA axis [8]. The surgical removal of the adrenal gland (adrenalectomy) has been widely used in animal research [16]. In the current study, adrenalectomy in addition to guanethidine at the dose effective to block adrenergic terminals were observed to attenuate the hyperglycemia caused by liraglutide [22]. This met the criteria for suggesting that liraglutide causes hyperglycemia in normal rats via sympathoadrenal activation, as previously described [8]. However, liraglutide reduced hyperglycemia in type 1 diabetic rats also by activating the adrenal gland [11]. Thus, we investigated the difference in the adrenal gland between rats with and without diabetes. Notably, liraglutide activates the adrenal glands isolated from type 1 diabetic rats at the dose that did not modify the adrenal glands isolated from normal rats using the secretion of beta-endorphin as an indicator. Changes in GLP-1R mRNA levels backed up this view. There is no doubt that adrenal gland activation in normal rats is primarily controlled by the brain via the HPA axis [18]. It has also been shown that the HPA axis is activated in type 1 diabetic rats [23]. However, liraglutide stimulated the adrenal gland before influencing the HPA axis in type 1 diabetic rats. Although more research is required, the affinity of GLP-1R in the adrenal gland raised by hyperglycemia seems possible.

The current study discovered liraglutide’s glucose-increasing effect in normal rats and demonstrated it to be the same as Ex-4, another GLP-1R ligand, by activating the HPA axis in the brain. GLP-1R agonists have been shown in clinical trials to have fewer side effects on hypoglycemic risk [6]. This appears to be associated with the current findings, since GLP-1R activation may activate the sympathetic nervous system, resulting in an acute increase in blood glucose levels [10,17]. However, more research appears to be required in the future.

The limitations were related to the primary goal of focusing solely on the effect of liraglutide. Agonists of GLP-1R used in clinics will be compared ahead of time. More evidence, including Western blot or immunohistochemistry, is needed to better understand the molecular mechanism of liraglutide in the adrenal glands. Dulaglutide is a long-acting GLP-1R agonist that is well tolerated as monotherapy or as an add-on therapy in clinics [24]. Dulaglutide has been shown to reduce cognitive impairments in people (aged 50 years) with type 2 diabetes [25] and to inhibit ethanol intake in animals [26], indicating that it can enter the brain easily. In healthy volunteers, however, dulaglutide at 1.5 mg once weekly failed to alter the HPA axis [9]. Furthermore, lixisenatide, like liraglutide, is an agonist that can enter the brain and activate the GLP-1R [27]. The effects of lixisenatide and other agonists, such as albiglutide and oral semaglutide, on the HPA axis are still unknown. Therefore, further research into the activation of the HPA axis by GLP-1 analogue(s) is required in the future.

## 4. Materials and Methods

### 4.1. Materials

Liraglutide was purchased from Novo Nordisk (Bagsvaerd, Denmark). Exendin 9–39 (Ex9), Exendin-4 (Ex-4), guanethidine, and other chemicals and reagents were purchased from Sigma-Aldrich (St. Louis, MO, USA).

### 4.2. Animal Model

A total of 120 male Sprague Dawley (SD) rats weighing 260 to 290 g were supplied by the National Laboratory Animal Center (Taipei, Taiwan). Rats were acclimated to the housing facility for one week after arrival before the study procedures began. We induced the diabetic model in overnight fasted rats through an intravenous injection of STZ (65 mg/kg) according to our previous method [18]. One week after STZ treatment, the rats with fasting blood glucose levels over 300 mg/dL were characterized as diabetic. Then, they were used for experiments within 2 weeks. All rats were anaesthetized with intraperitoneal injection of sodium pentobarbital (35 mg/kg) to minimize the suffering before the procedures. The experimental procedures in the current study using animals have been approved by the Institutional Animal Ethics Committee of Chi-Mei Medical Center (107101701) and were performed in accordance with the NIH Guide for the Care and Use of Laboratory Animals.

### 4.3. Laboratory Determinations

Blood samples of rats were collected from the tail vein. Following centrifugation, blood plasma was separated from blood cells to estimate the plasma glucose and plasma biomarker levels. Plasma glucose levels determined by the glucose oxidase method, using an analyzer (Quik-Lab, Ames; Miles Inc., Elkhart, Indiana), were performed by our previous method [6]. Biomarkers in plasma levels were measured by enzyme-linked immunosorbent assay (ELISA) using a commercial kit, including adrenocorticotropic hormone (ACTH, Cat. No. A7505, Sigma-Aldrich, St. Louis, MO, USA), cortisol (Cat. No. SE120082, Sigma-Aldrich, St. Louis, MO, USA), and beta-endorphin (Cat. No. S-1352, Peninsula Laboratories, Belmont, CA, USA).

Rats were housed in individual cages for food intake measurements, and food and tap water were provided ad libitum. The rats were given a set amount of food so that the first intake measurements could be taken the next day.

### 4.4. Adrenalectomy in Rats

Rats under anesthesia with sodium pentobarbital (35 mg/kg i.p.) received bilateral adrenalectomy through the dorsal approach following a previous report [18]. Rats receiving the controls were performed in same manner. All animals were in good health after the surgery. Adrenalectomized rats received saline supplemented with corticosterone to replace the drinking water for 2 weeks and then supplied the saline alone (gradually reduced within a week). After recovery, adrenalectomized rats were used to induce diabetes or not.

### 4.5. Incubation of Isolated Adrenal Gland

Adrenal glands isolated from the sacrificed rats with diabetes or not were used to remove the cortex immediately following our previous method [28]. Tissues in 2 mL modified Krebs solution underwent 15 min of pre-incubation at 37 °C in an incubator bubbled with air (95% O_2_ and 5% CO_2_) under continuous agitation, as described in our previous report [28]. Then, the tissues were moved to fresh tubes to incubate with liraglutide for another 60 min under the same conditions. The tubes were placed on ice and used to terminate the reaction. Incubated medium was immediately collected and then frozen at −70 °C until the assay of beta-endorphin-like immunoreactivity (BER) [18].

### 4.6. Intracerebroventricular (ICV) Injection

Rats under anesthesia with pentobarbital sodium (35 mg/kg, i.p.) were placed in a stereotaxic apparatus (WPI Ltd., Hertfordshire, UK). The scalp was incised, and the skull was levelled off around the bregma. Following our previous methods [29], a 22-gauge, 12 mm stainless-steel guide cannula was inserted in the right lateral ventricle of the brain. The third cerebroventricular area was targeted in rats using the following coordinates: −1.0 mm anteroposterior, 0 mm mediolateral, and −7.5 mm dorsoventral as described previously [9]. Three screws and dental acrylic were used to secure the cannula to the skull. Furthermore, a stainless tube (0.5 mm protrusion) was left inside the cannula to prevent blood clots from blocking the cannula. During a 7-day recovery period following surgery, rats were given ampicillin (1 mg/kg, i.p.) daily to prevent infection. ICV injections of liraglutide or normal saline (vehicle control) were then used to inject 1 μL of the testing solution into the lateral ventricle using an injection needle inserted into the guide cannula.

### 4.7. Quantitative Reverse-Transcription Polymerase Chain Reaction (qRT-PCR)

TRIzol was used to isolate total RNA from the adrenal medulla. Roche provided the primers used in qRTPCR (Roche Diagnostics GmbH, Mannheim, Germany). Each PCR product’s concentration was calculated in relation to a corresponding standard curve. Realtime quantitative PCR and the 2-ΔΔCq method were used to analyze gene expression data. Then, as described in the previous report [30], each gene’s expression was calculated as the ratio of target gene level to that of β-actin, the housekeeping control. Duplicated assay was run for each sample. The used primers were as follows: forward: 5′-AGTGCGAAGAGTCCAAGCAA-3′ and reverse: 5′-TTGAGGGCAGCGTCTTTGAT-3′ (GLP-1 Receptor), forward: 5′-CATCCAGGCTGTGTTGTCCC-3′ and reverse: 5′-CACGCACGATTTCCCTCTCA-3′ (β-actin).

### 4.8. Statistical Analysis

Results subjected to statistical analysis using SPSS 23 (Armonk, NY, USA) were indicated as means ± SEM in each group. They were analyzed using one-way ANOVA and Dunnett’s post hoc test, or two-way ANOVA and Bonferroni’s post hoc tests. The normal distribution of the data was checked using the maximum likelihood method, with the significance level set at *p* < 0.05.

## 5. Conclusions

The current study discovered that liraglutide may activate the HPA axis in the brain, increasing plasma glucose in normal rats after a single treatment. Tolerance to this hyperglycemia was observed in normal rats given a daily injection for one week or more. Notably, normal rats demonstrated cross-tolerance between liraglutide and Ex-4. Liraglutide, on the other hand, has been shown to reduce hyperglycemia in type 1 diabetic rats by activating the GLP-1R in the adrenal gland. The current study discovered that liraglutide influenced the adrenal gland before entering the brain in diabetic rats, indicating that hyperglycemia caused a change in GLP-1R sensitivity. Therefore, whether the rats have diabetes or not, the effect of liraglutide on glycemic regulation differs.

## Data Availability

Data is contained within the article.
